# Mirror feedback in Zumba dance education: effects on proprioception, balance and mood state

**DOI:** 10.7717/peerj.20453

**Published:** 2026-02-26

**Authors:** Gizem Ceylan Acar, Semiyha Tuncel

**Affiliations:** 1 Faculty of Sport Sciences, Department of Sports Management, Muş Alparslan University, Muş, Türkiye; 2 Faculty of Sports Sciences, Department of Physical Education and Sports, Ankara University, Ankara, Türkiye

**Keywords:** Mirror, Dance, Mood, Training, Joint position sense, Static-dynamic balance

## Abstract

**Background:**

This study aims to investigate the effects of Zumba dance training conducted in a mirrored environment on participants’ knee joint position sense (proprioception), static-dynamic balance and mood state.

**Methods:**

A total of 39 female volunteers aged 18 to 25 participated in the study. Participants were randomly and counterbalanced assigned to three groups: mirror Zumba training group (*n* = 14), non-mirror Zumba training group (*n* = 13), and a control group (*n* = 12). Zumba sessions were held for 10 weeks, three times per week, one hour per session. Data collection took place at Ankara University, Faculty of Sports Sciences, Performance Laboratory. Participants visited the laboratory four times and underwent assessments for body composition (body mass index (BMI)), knee joint position sense, static and dynamic balance, and mood using the Positive and Negative Affect Schedule (PANAS).

**Results:**

Zumba training performed in front of mirrors significantly impaired knee joint proprioception (*p* < .05). The mirror group showed reduced proprioceptive sensitivity at 15°, 16 30°, 60°, and 90° knee angles, whereas the non-mirror group demonstrated significant improvement at the same angles. Mirror use also negatively affected static balance while facilitating dynamic balance. However, no significant differences were found in mood states between groups, indicating that mirror presence did not impact emotional well-being.

**Conclusions:**

The use of mirrors during Zumba dance training may disrupt proprioceptive development and static balance, although it can enhance dynamic balance. Since mirror use did not influence mood, its impact appears primarily physical. These results suggest that mirrors in group exercise settings should be used selectively, especially when the goal is to enhance proprioception.

## Introduction

Dance genres are often considered part of the sports field due to their performance-based nature (Wanke et al., 2014). In this context, Zumba is a dance-based exercise widely practiced as one of the most popular group classes in fitness centers (Delextrat et al., 2016). Mirrors, frequently used in group exercises such as Zumba, Fitness, Pilates, Yoga, Martial Arts, and Weight Training, serve primarily as visual feedback tools. Although the inclusion of mirrors in training environments began historically with ballet in the late 18th century (Ehrenberg, 2010), they have not been systematically adopted as pedagogical instruments in classical or contemporary dance techniques (Brown & Meulenbroek, 2016).

In recent years, educators and researchers have questioned the pedagogical value of learning dance skills by observing oneself in a mirror (Krasnow & Wilmerding, 2015). Despite limited research, some studies suggest that mirrors, as a form of external feedback, may facilitate motor learning, improve coordination, and enhance long-term skill retention (Dearborn et al., 2006; Weber & Didier, 2021). However, other findings indicate potential negative psychological outcomes, such as decreased body satisfaction, altered mood states, reduced self-efficacy, and increased social anxiety (Radell et al., 2011; Martin Ginis, Jung & Gauvin, 2003; Frayeh & Lewis, 2018).

Mirrors have also been examined in the context of balance training, particularly in disciplines like gymnastics and dance (Notarnicola et al., 2014; Miller et al., 2018). While balance training is known to support proprioceptive development (Hanney, 2000; Hutt & Redding, 2014), some researchers argue that excessive reliance on visual cues can inhibit attention to kinesthetic signals, thus hindering proprioceptive awareness (Brodie & Lobel, 2008; Diehl, 2016). Since balance relies on integrating visual, vestibular, and proprioceptive inputs (Daneshjoo et al., 2012), mirror feedback may sometimes disrupt this sensory balance.

Given the interrelated nature of body image, proprioception, and performance in technical skill development (Radell, Adame & Cole, 2002), mirror use may have both facilitating and distracting effects. It has been suggested that mirrors can impair concentration and limit the use of kinesthetic and proprioceptive senses during movement execution (Radell et al., 2014). Proprioception, defined as the ability to perceive the body’s position and movement, plays a critical role in athletic performance and physical well-being (Yılmaz et al., 2024). While mirrors undoubtedly provide immediate visual feedback about posture and movement alignment, this feedback may overshadow proprioceptive processing in visually dominant learning environments (Oliver, 2008).

Zumba, a holistic exercise format combining aerobic movement and dance elements, requires the active engagement of motor skills such as balance, coordination, postural control, and proprioceptive awareness (Donath et al., 2014; Delextrat et al., 2016). Due to its rhythm-driven and direction-changing movements, Zumba particularly challenges lower extremity control, making joint position sense and dynamic balance critically important (Steinberg et al., 2015). As a group activity often performed in mirrored environments, it also influences participants’ body perception, attentional focus, and emotional states (Radell et al., 2014; Frayeh & Lewis, 2018). Therefore, examining proprioception, balance, and mood together in the context of Zumba skill acquisition provides a comprehensive perspective on motor learning while also offering insight into the pedagogical implications of visual feedback (Dearborn & Ross, 2006; Brodie & Lobel, 2008). Moreover, Zumba training has been shown to positively influence not only motor performance but also psychosocial variables such as mood and health-related quality of life (Waer et al., 2024a; Waer et al., 2024b; Waer et al., 2024c), highlighting its holistic benefits on both physical and emotional well-being. Recently, Zumba training has been shown to yield not only physical but also psychological benefits. Recent evidence also supports these findings. Waer et al. (2024a), Waer et al. (2024b) and Waer et al. (2024c) compared the effects of Zumba and Pilates training in postmenopausal women and found that Zumba produced greater improvements in postural control, balance, mood, and quality of life, even under conflicting sensory conditions. These results highlight Zumba’s dual benefits for both physical and emotional dimensions of well-being.

Although some quantitative studies have investigated the effect of mirrors on static and dynamic balance (Coker, 2016; Miller et al., 2018), there is no consensus in the literature regarding their specific impact on proprioceptive abilities. Therefore, this study aims to investigate the effects of consciously and systematically implemented Zumba dance training in a mirror environment on participants’ knee joint position sense (proprioception), static and dynamic balance, and mood state. It is hypothesized that mirror use will negatively affect joint position sense and dynamic balance, while having a positive effect on static balance. Furthermore, mirror use is expected to have no significant effect on mood state.

## Methods

### Participants

A total of 39 women who were studying at Ankara University, Faculty of Sport Sciences, aged between 18–25 years (Table 1), had not engaged in regular physical activity in the last 6 months, had not experienced any musculoskeletal injuries in the last 1 year and had not received zumba or dance training before participated in this study voluntarily. Exclusion criteria from the study included: experiencing any discomfort or health issues during the study, participating in any exercise program outside the scope of the study, non-compliance with the prescribed training program (*e.g.*, irregular attendance), and the participant’s voluntary decision to withdraw from the study.

Although the participants were students at the Faculty of Sport Sciences, they were classified as physically inactive individuals based on the fact that they had not engaged in any regular exercise program in the past six months. This study was conducted in the fall semester of the 2021–2022 academic year, immediately after the 2020–2021 academic year—a period during which universities transitioned to remote education due to the COVID-19 pandemic, gyms were closed, and lockdown measures were in effect. Therefore, it is known that the participants had not taken part in any practical courses, either individually or within the curriculum, during that period. However, considering the faculty curriculum includes certain practical courses (*e.g.*, basic training or sport-specific technique classes), it was acknowledged that students might occasionally have been exposed to low- to moderate-intensity physical activity. Nevertheless, to ensure homogeneity between groups in terms of shared academic background, motor experience, and physical competence, this variable was consciously controlled. Furthermore, the fact that the control group also participated in practical physical education courses of similar structure and intensity allowed for the isolation of the effects of the Zumba training conducted in a mirrored environment from those of general physical activity. The control group was included to allow for a comparative evaluation of the intervention’s effects and continued with regular academic activities without receiving any dance training. This design enabled the differentiation of natural time-based changes and external influences.

This study was conducted in compliance with the ethical principles stated in the Declaration of Helsinki and ICH Good Clinical Practice guidelines. Ankara University Faculty of Medicine Clinical Research Ethics Committee approval (Approval no: I10-602-20, Date: 12.11.2020) was obtained for the study and an “Informed consent form” was completed by all participants.

**Table 1:** Sample means and standard deviations for demographic measures.

	**Mirror zumba group (*n* = 14)**	**Non-mirror zumba group (*n* = 13)**	**Control group (*n* = 12)**
**Variables**	**Mean ± SD**	**Mean ± SD**	**Mean ± SD**
Age (years)	20.00 ± 1.46	20.69, ± 3.32	19.66 ± 1.23
Height (cm)	161.80 ± 8.67	162.18 ± 4.58	164.19 ± 5.96
BMI (kg/m^2^)	20.92 ± 2.67	21.67 ± 2.26	20.86 ± 3.92

**Notes.**

BMI Body mass index SD standard deviation

### Study design

**Figure 1:**
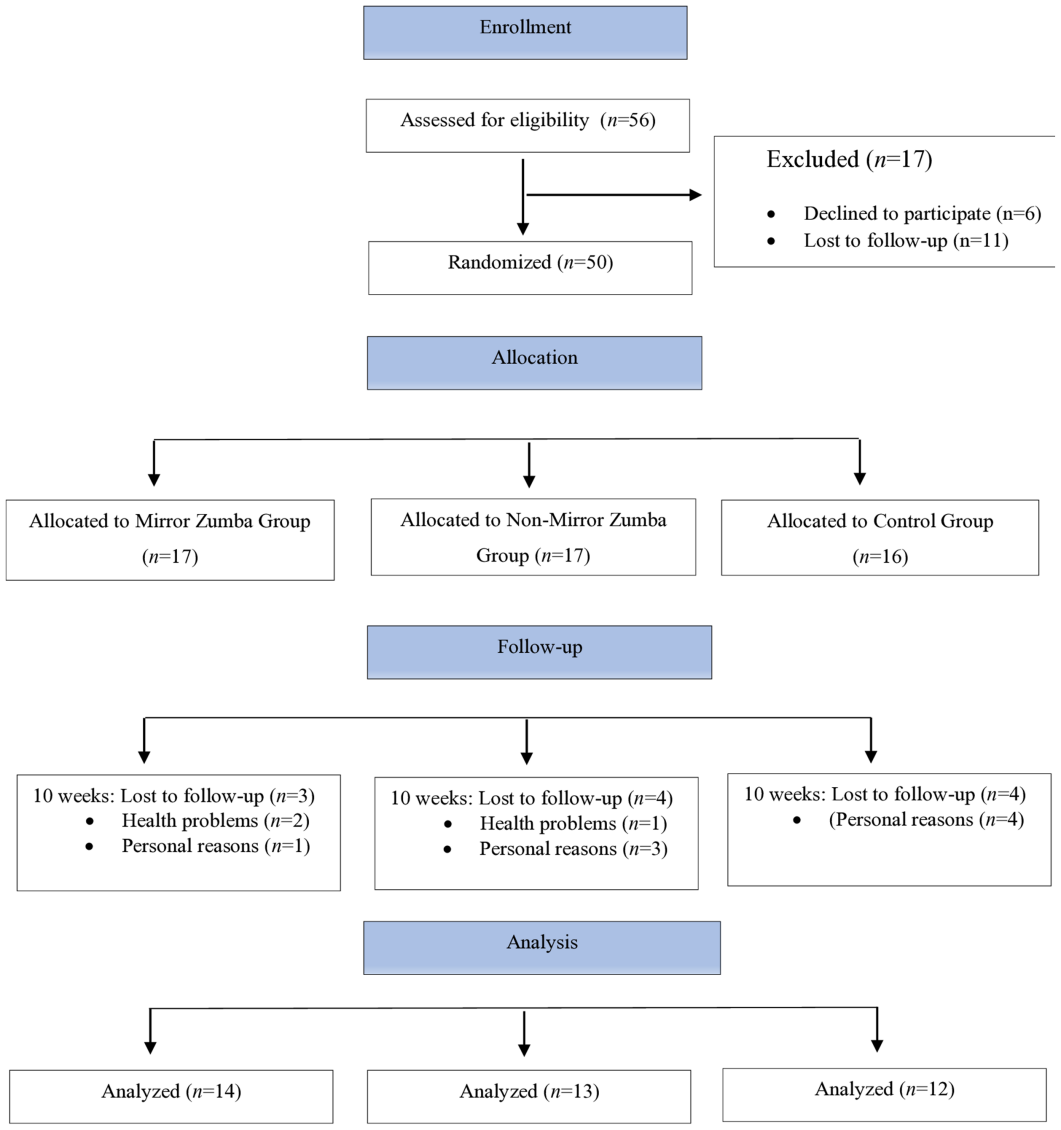
Study design and participant flowchart.

In the study, participants were randomly and counterbalanced assigned into three groups: a Zumba training group in a mirrored studio (*n* = 14), a Zumba training group in a non-mirror studio (*n* = 13), and a control group that did not receive any dance training (*n* = 12). Both experimental groups received the same Zumba training content, delivered by the same instructor (Fig. 1). The training sessions were conducted for 10 weeks, 3 days per week, 1 h per day. All tests and measurements were carried out at the Performance Laboratory of the Faculty of Sport Sciences, Ankara University. Before the intervention, participants were informed about the study procedures and informed consent and personal information forms were collected. Measurements were performed at four time points: Week 1 (pre-test), Week 5 (mid-test), Week 10 (post-test), and Week 15 (retention test). The Positive and Negative Affect Schedule (PANAS) was administered twice, at the pre-test and post-test stages. PANAS was not administered during the intermediate and follow-up testing phases; this was due to a preference to reduce participant burden and to focus primarily on motor and proprioceptive variables. The assessed variables included: body mass index (BMI), knee joint position sense, and static and dynamic balance. All participants were familiarized with the testing protocols one day before the first measurement, including a low-intensity practice session to ensure understanding of the procedures.

### Procedures

Participants were instructed to refrain from consuming any food or beverages (except water) within four hours before the measurements and to avoid alcohol, diuretic products, and any physical activity for at least 12 h before testing. Additionally, it is recommended that participants should be hungry, have an empty bladder and not wear any metal jewellery before the pre-assessment to increase the accuracy of the measurements. Especially in bioimpedance analysis (BIA), changes in body fluid balance (*e.g.*, eating, urine accumulation) can directly affect the measurement results (Kyle et al., 2004). Similarly, in balance tests, metal jewellery or accessories can impair the accuracy of body movement tracking sensors or visual balance measurement tools (Ruhe, Fejer & Walker, 2010). Therefore, the relevant pretest protocols are scientifically based. Body height was measured barefoot and in an upright posture using a Harpenden Stadiometer (Holtain, U.K.), while body weight and composition were assessed using the PlusAvis 333 analyzer (Jawon Medical, South Korea). Knee joint position sense was measured using a Saunders Digital Inclinometer. Participants were first shown target knee flexion angles of 15°, 30°, 60°, and 90° with their eyes open and were then asked to replicate these angles with their eyes closed for both the right and left legs. Each angle was repeated three times, and the most accurate measurement was recorded. Balance assessments were conducted using the Biodex System SD (Biodex Medical Systems, Shirley, NY) and included the Postural Stability Test (PST) and the Athletic Single Leg Test (ASL). All tests were performed with both eyes open and eyes closed, but only the data collected with eyes closed were included in the analysis. Static balance testing was carried out on a fixed platform, while dynamic balance testing was conducted on a movable platform set at difficulty level 6 to reflect fall risk. Each test consisted of three trials of 20 s with 10-second rest intervals between trials. The balance parameters evaluated included the overall stability index (OSI) for both static and dynamic conditions (S-OSI, D-OSI), the Anterior-Posterior Stability Index (S-API, D-API), and the Medial-Lateral Stability Index (S-MLI, D-MLI).

### The positive and negative affect schedule

PANAS, developed by Watson, Clark & Tellegen (1988) and adapted into Turkish by Gök, Selçuk & Gençöz (2018) was used to assess the participants’ mood states. The scale consists of 20 items, with 10 items measuring positive affect (*e.g.*, excited, strong, attentive) and 10 measuring negative affect (*e.g.*, distressed, irritable, afraid). Each item is rated on a 5-point Likert scale ranging from 1 = very slightly or not at all to 5 = extremely. The internal consistency coefficients previously reported for the Turkish version were *α* = .86 for the positive effect and *α* = .83 for the negative effect. In the present study, the reliability coefficients were recalculated as *α* = .89 for the positive affect subscale and *α* = .81 for the negative affect subscale. The PANAS was administered only at the pre-test and post-test measurement points.

### Zumba dance training

The researcher attended Zumba instructor trainings between 2017 and 2020 in order to personally teach Zumba sessions to both the mirrored and non-mirrored groups and to improve her skills in this area. Through these trainings, she enhanced her understanding of the difficulty levels and teaching techniques of choreographies designed for beginners. The sessions were conducted in a mirrored dance studio with adequate lighting and ventilation at the Faculty of Sport Sciences, Ankara University. As regular attendance was a requirement, participant absenteeism was recorded, and adherence to at least 85% of the total sessions was ensured. This approach aimed to facilitate the correct instruction of movements and to maintain safety. Participants were encouraged to exercise at a self-selected intensity; however, their perceived exertion levels (RPE) were informally monitored throughout the sessions.

The overall exercise intensity was regulated through the tempo of the music, which directly influenced the pace and complexity of the movements. This approach corresponds to moderate-to-vigorous intensity levels and is consistent with standard Zumba practice, where music tempo is used to progressively increase cardiovascular and muscular workload.

The intensity of the Zumba exercises implemented in the study was determined based on music tempo. The warm-up phase included basic non-jumping dance steps (*e.g.*, march, step touch, side to side) accompanied by music with a tempo ranging from 120 to 140 beats per minute (bpm), lasting approximately 8–10 min. The main workout phase was conducted using 8 to 10 original Zumba songs with tempos between 140 and 160 bpm. Each dance choreography lasted 3–5 min, with short rest intervals of 15–30 s between dances. The choreography was structured to progress from simple to complex movements over the weeks and was taught to participants in that order. The movements were designed to engage both upper and lower extremities, including the feet, knees, hips, trunk, arms, and hands. While the choreography was primarily based on Latin dance steps, it also incorporated functional fitness exercises such as squats, lunges, lunge jumps, lateral lunges, planks, knee lifts, cross-body knee lifts, and jumping exercises. At the end of each session, a cool-down and stretching segment was performed using low-tempo music. The training sessions were structured to promote cardiovascular activity through dance while supporting rhythm and coordination development.

### Statistical analysis

All data were analyzed using IBM SPSS Statistics 22.0 (IBM Corp., Armonk, NY, USA). Results were presented as mean ± standard deviation, and the level of significance was set at *p* < 0.05. The normality of the data distribution was assessed using the Shapiro–Wilk test. To determine differences between groups and across time points, a repeated measure mixed analysis of variance (Mixed ANOVA) was performed. The assumption of sphericity was tested using Mauchly’s test. In cases where the assumption of sphericity was violated, the Greenhouse-Geisser correction (*ɛ* < 0.75) or the Huynh-Feldt correction (*ɛ* > 0.75) was applied based on epsilon values. Pairwise comparisons between measurement points were conducted using the Bonferroni post-hoc test. Additionally, effect sizes were calculated using partial eta squared (*η*^2^p), with *η*^2^ = 0.01 considered small, *η*^2^p = 0.06 medium, and *η*^2^p ≥ 0.14 considered large (Richardson, 2011).

## Result

A total of 39 women were included in this study. The descriptive data of the participants are presented in Table 1.

**Figure 2:**
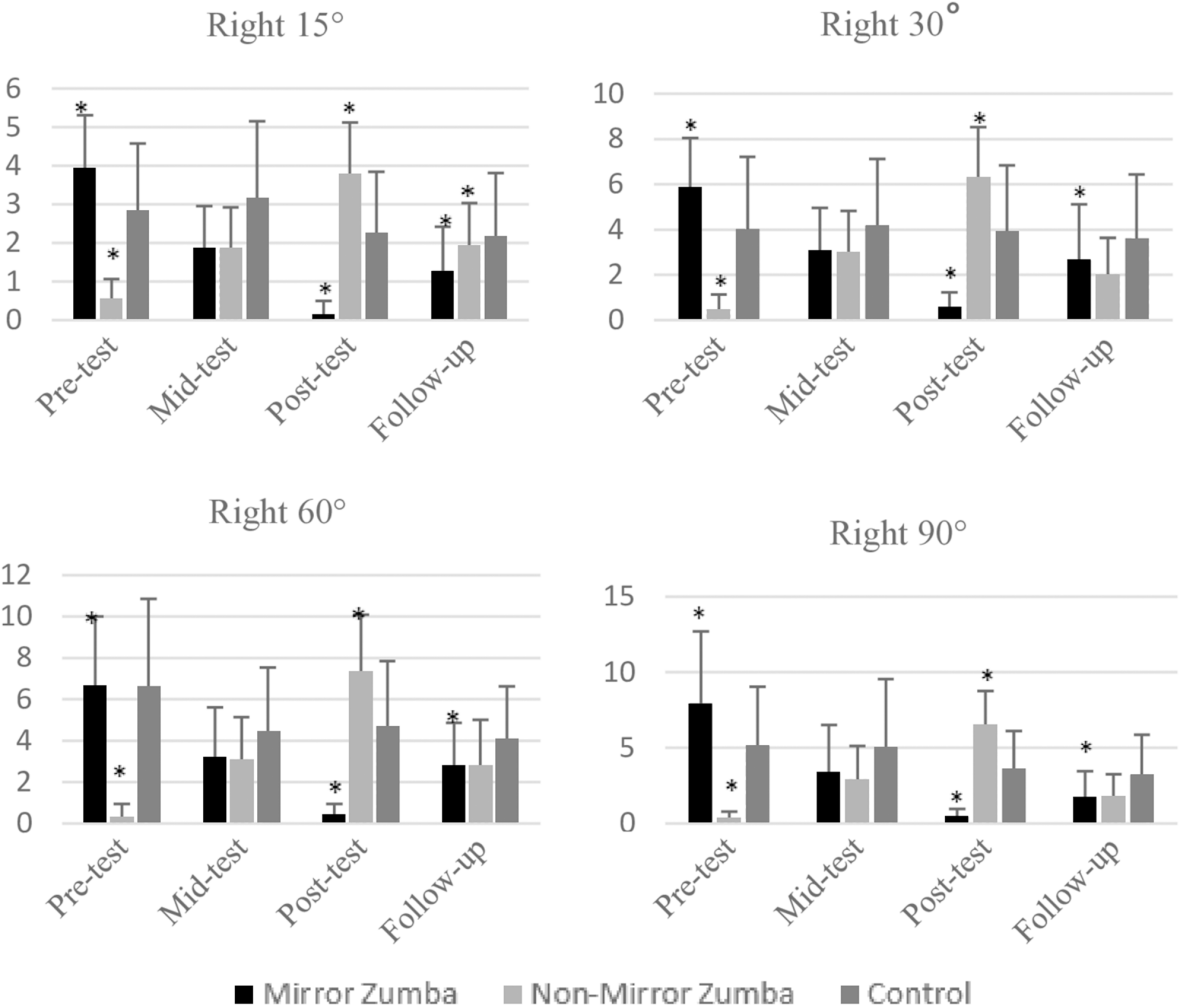
Right knee 15°–30°–60°–90° angle inclinometer measurements of mirror group, non-mirror group, and control group. 15° time main effect (*F*_3.108_ = 3.070; *p* = 0.031; *η*^2^p = 0.079) and time × group interaction (F_6.108_ = 28.656; *p* = 0.001; *η*^2^ p = 0.614); 30° time main effect (F_3.108_ = 1.960; *p* = 0.123; *η*^2^p = 0.052) and time × group interaction (F_6.108_ = 24.798; *p* = 0.001; *η*^2^p = 0.579); 60° time main effect (F_3.108_ = 3.417; *p* = 0.020; *η*^2^p = 0.087) and time × group interaction (F6.108 = 26.789; *p* = 0.001; *η*^2^p = 0.598); 90° time main effect (F_3.108_ = 7.241; *p* < 0.001; *η*^2^p = 0.167) time × group interaction (F_6.108_ = 23.367; *p* < 0.001; *η*^2^p = 0.565). An asterisk (*) indicates a statistically significant difference (*p* < 0.05) between the compared groups.

In the joint position sense measurements for the right knee, both time main effect and time × group interaction were found to be statistically significant at 15°, 60°, and 90° angle positions. Only the time × group interaction is significant in the 30° angle position. This reveals that in some respects there is a general development over time, but also significant differences between groups. When intragroup analyses were evaluated: In the mirror group, a significant decrease was observed between the pre-test and post-test in all angles (*p* = 0.001); this decrease was also supported by the follow-up test in all angle positions. This finding suggests that mirrored applications may produce both effective and permanent results in proprioceptive development. In the non-mirror group, although there was a significant increase between the pre-test and post-test in general (*p* = 0.001), it was observed that these improvements were not fully sustained in the follow-up test and did not reach statistical significance, especially at 30° and 90° angles. This indicates that the non-mirror learning process may be limited in terms of permanence. No significant change was detected in any angle position in the control group (*p* > 0.05) (See Fig. 2).

**Figure 3:**
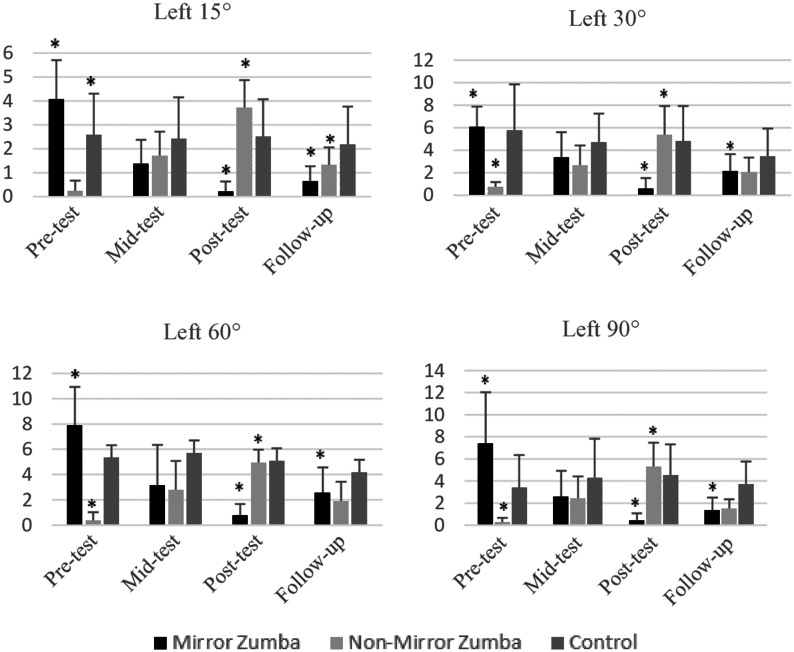
Left knee 15°–30°–60°–90° angle inclinometer measurements of mirror group, non- mirror group, and control group. 15° time main effect (F_3.108_ = 7.361; *p* = 0.001; *η*^2^p = 0.170) and time × group interaction (F_6.108_ = 35.787; *p* = 0.001; *η*^2^p = 0.665); 30° time main effect (F_3.108_ = 9.298; *p* = 0.001; *η*^2^p = 0.205) and time × group interaction (F_6.108_ = 29.846; *p* = 0.001; *η*^2^p = 0.624); 60° time main effect (F_3.108_ = 5.018; *p* = 0.003; *η*^2^p = 0.122) and time × group interaction (F_6.108_ = 21.535; *p* = 0.001; *η*^2^p = 0.545); 90° main effect of time (F_3.108_ = 4.086; *p* = 0.005; *η*^2^p = 0.102) and time × group interaction (F_6.108_ = 21.544; *p* = 0.001; *η*^2^p = 0.545). An asterisk (*) indicates a statistically significant difference (*p* < 0.05) between the compared groups.

In the joint position sense measurements for the left knee, both the time main effect and the time × group interaction were found to be statistically significant at 15°, 30°, 60° and 90° for all angle positions. These findings suggest that the intervention had significant effects on different groups in a time-dependent manner. By intragroup analyses: In the mirror group, a significant decrease was observed between the pre-test and the post-test in each angle position (*p* = 0.001) and this change in the direction of decrease was supported by the follow-up test results, indicating its permanence. In the non-mirror group, significant increases were found in general (*p* = 0.001), but this increase was not statistically confirmed in other angle positions except for the results of the 15° follow-up test. In the control group, no significant change was found in any angle position; this supports that the observed changes were due to the intervention (*p* > 0.05) (see Fig. 3). The significant improvements observed in the non-mirror group in the short term were not statistically sustained in the follow-up test, especially in the 15° angle position. This finding suggests that although non-mirror learning is initially effective, its effect diminishes over time. On the other hand, statistically significant decreases in both the post-test and the follow-up test in the mirror group revealed that the learned skill became more permanent. Therefore, it can be said that mirror practices offer advantages not only in terms of instant improvement but also in terms of learning transfer and retention.

### Static and dynamic balance measurements results

#### Postural Stability Static Balance

There was no main effect of time on the OSI variable of the PST eyes closed (EC) static balance measurement (*F*_3.108_ = 0.631; *p* = 0.597; *η*^2^p = 0.17), but a time ×group interaction was observed (*F*_6.108_ = 14.251; *p* = 0.001; *η*^2^p = 0.442). The source of the difference was evaluated by Bonferroni *Post-Hoc* analysis with a significance level of 0.05: It was determined that the EC static balance OSI value increased significantly (*p* = 0.001) between the pre- and post measurements of the mirror group in the group × time interaction, whereas there was no significant difference between the follow-up test and the pre-test (*p* > 0.05). It was found that the EC static balance OSI value decreased between pre- and post-measurements (*p* = 0.001) and there was a statistically significant difference in the group × time interaction of the non-mirror group (Pre-test (Pre): 1.42 ± 0.83; Follow-up (Fu): 0.97 ± 0.27); and when the measurements made during the follow-up week were compared with the pre-test, a statistically significant difference was found (*p* = 0.025). There was no time main effect of the PST EC static balance measure on the anterior posterior stability index (API) variable (*F*_3.108_ = 0.693; *p* = 0.558; *η*^2^p = 0.19). However, a time ×group interaction was observed (*F*_6.108_ = 5.312; *p* = 0.001; *η*^2^p = 0.228). The source of the difference was evaluated by Bonferroni *Post-Hoc* analysis with a significance level of 0.05: It was determined that there was no significant difference (*p* > 0.05) between the pre-test and post-test measurements of the EC static balance API variable of the mirror group and between the follow-up test and the pre-test. It was determined that the EC Static balance API value decreased between pre and post-measurements in the group ×time interaction of the group without mirror (*p* = 0.009), but there was no significant difference between the follow-up test and the pre-test (*p* > 0.05). No main effect of time for the Medial-Lateral Stability Index (MLI) variable of the PST EC static balance measurement was observed (*F*_3.108_ = 1.156; *p* = 0.330; *η*^2^p = 0.031). However, a time × group interaction was observed (*F*_6.108_ = 4.562; *p* < 0.001; *η*^2^p = 0.202). The source of the difference was evaluated by Bonferroni *Post-Hoc* analysis with a significance level of 0.05: It was determined that there was no significant difference (*p* > 0.05) between the pre-test and post-test measurements of the EC static balance MLI variable of the mirror group and between the follow-up test and the pre-test. It was determined that the EC Static balance MLI value decreased between pre and post-measurements in the group × time interaction of the group without mirror (*p* = 0.001), but there was no significant difference between the follow-up test and the pre-test (*p* > 0.05). In the control group, no significant change was observed in all measurements (OSI-API-MLI) (*p* > 0.05) (Table 2).

**Table 2:** Postural Stability (PST) Static Balance Test and Postural Stability (PST) Dynamic Balance Test.

**Eyes closed**		**Mirror zumba group**	**Non-Mirror zumba group**	**Control group**	**Postural Stability (PS) Static Balance Test**			
		**Mean ± SD**	**Mean ± SD**	**Mean ± SD**	**Group**	**F**	***p***	*η* ^**2**^ **p**
	Pre-test	1.05 ± 0.43	1.41 ± 0.82	1.48 ± 1.09				
	Mid-test	1.6 ± 0.73	0.7 ± 0.22	1.54 ± 1.27	^a^	0.631	0.597	0.17
OSI	Post-test	1.87 ± 0.84^*+^	0.51 ± 0.15^*-^	1.48 ± 1.07	^b^	14.251	**0.001**	0.442
	Follow-up	1.16 ± 0.4	0.96 ± 0.26^*-^	1.49 ± 1.05				
	Pre-test	1.06 ± 0.91	1.03 ± 0.89	0.57 ± 0.34				
	Mid-test	0.5 ± 0.16	1.2 ± 1.21	0.77 ± 0.66	^a^	0.693	0.558	0.19
API	Post-test	0.34 ± 0.12	1.37 ± 1.06^*-^	0.9 ± 0.64	^b^	5.312	**0.001**	0.228
	Follow-up	0.65 ± 0.24	1.23 ± 1.04	0.59 ± 0.29				
	Pre-test	0.57 ± 0.34	0.73 ± 0.36	0.62 ± 0.55				
	Mid-test	0.77 ± 0.66	0.4 ± 0.23	0.61 ± 0.63	^a^	1.156	0.330	0.031
MLI	Post-test	0.9 ± 0.64	0.23 ± 0.15^*-^	0.38 ± 0.24	^b^	4.562	**0.001**	0.202
	Follow-up	0.59 ± 0.29	0.6 ± 0.29	0.65 ± 0.23				

					**Postural Stability (PS) Dynamic Balance Test**			
	Pre-test	2.68 ± 0.89	1.19 ± 0.45	3.05 ± 1.83				
	Mid-test	2.12 ± 0.6	2.46 ± 1.13	2.95 ± 1.84	^a^	1.283		0.284	0.34
OSI	Post-test	1.27 ± 0.51^*-^	2.99 ± 1.22^*+^	3.24 ± 2.04	^b^	22.737		**0.001**	0.558
	Follow-up	1.9 ± 0.4^*-^	2.25 ± 0.87^*-^	3.23 ± 1.84				
	Pre-test	1.83 ± 0.66	0.83 ± 0.39	2.29 ± 1.87				
	Mid-test	1.65 ± 0.63	1.67 ± 0.96	1.18 ± 0.54	^a^	0.274		0.844	0.008
API	Post-test	0.89 ± 0.35^*-^	1.13 ± 0.92^*+^	2.3 ± 1.84	^b^	8.789		**0.001**	0.328
	Follow-up	1.48 ± 0.34	1.44 ± 0.65^*-^	2.29 ± 1.36				
	Pre-test	1.6 ± 0.75	0.62 ± 0.23	1.53 ± 0.7				
	Mid-test	0.95 ± 0.43	1.22 ± 0.44	1.41 ± 1	^a^	2.538		0.060	0.066
MLI	Post-test	0.71 ± 0.41^*-^	1.82 ± 0.66^*+^	1.86 ± 0.75	^b^	12.605		**0.001**	0.412
	Follow-up	0.92 ± 0.42^*+^	1.36 ± 0.63^*-^	1.8 ± 1.09				

**Notes.**

a Main effect of time.

b Time × Group interaction.

*+ Statistically significant positive change across pre-test, post-test, and follow-up test.

*- Statistically significant negative change across pre-test, post-test, and follow-up test.

OSI Overall Stability Index API Anterior–Posterior Stability Index MLI Medial–Lateral Stability Index

Mean and standard deviation values are presented for each variable. Bold styling indicates statistically significant values.

#### Postural stability dynamic balance test

No main effect of time for the OSI variable of the PST EC dynamic balance measurement was observed (*F*_3.108_ = 1.283; *p* = 0.284; *η*^2^p = 0.34). However, a time × group interaction was observed (*F*_6.108_ = 22.737; *p* < 0.001; *η*^2^p = 0.558). The source of the difference was evaluated by Bonferroni *Post-Hoc* analysis with a significance level of 0.05: It was found that the EC Dynamic balance OSI value decreased significantly (*p* = 0.001) between the pre- and post-measurements of the mirror group in the group × time interaction (*p* = 0.005) and there was a statistically significant difference when the measurements made in the follow-up week were compared with the pre-test (Pre: 2.69 ± 0.89; Fu: 1.90 ± 0.40). It was found that the EC Dynamic balance OSI value increased significantly (*p* = 0.001) between the pre- and post-measurements in the group × time interaction of the non-mirror group, and there was a statistically significant difference when the measurements made in the follow-up week were compared with the pre-test (Pre: 1.19 ± 0.46; Fu: 2.25 ± 0.87) (*p* = 0.001). No time main effect of PST EC Dynamic balance measurement on API variable was observed (*F*_3.108_ = 0.274; *p* = 0.844; *η*^2^p = 0.008). However, a time × group interaction was observed (*F*_6.108_ = 8.789; *p* < 0.001; *η*^2^p = 0.328). The source of the difference was evaluated by Bonferroni *Post-Hoc* analysis with a significance level of 0.05: It was determined that the EC Dynamic balance API value decreased significantly (*p* = 0.001) between the pre- and post-measurements of the mirror group in the group × time interaction, whereas there was no significant difference between the follow-up test and the pre-test (*p* > 0.05). It was found that the EC Dynamic balance API value increased significantly (*p* = 0.001) between the pre- and post-measurements in the group × time interaction of the non-mirror group and there was a statistically significant difference (*p* = 0.047) when the measurements made in the follow-up week were compared with the pre-test (Pre: 0.84 ± 0.39; Fu: 1.45 ± 0.66). No time main effect of PST EC Dynamic balance measurement on MLI variable was observed (*F*_3.108_ = 2.538; *p* = 0.060; *η*^2^p = 0.066). However, a time × group interaction was observed (*F*_6.108_ = 12.605; *p* < 0.001; *η*^2^p = 0.412). The source of the difference was evaluated by Bonferroni *Post-Hoc* analysis with a significance level of 0.05: It was found that the EC Dynamic balance MLI value decreased significantly (*p* = 0.001) between the pre and post measurements of the mirror group in the group × time interaction, and there was also a statistically significant difference (*p* = 0.011) when the measurements made in the follow-up week were compared with the pre-test (Pre: 1.62 ± 0.75; Fu: 0.93 ± 0.42). It was found that the EC Dynamic balance MLI value increased significantly (*p* = 0.001) between the pre- and post-measurements in the group × time interaction of the non-mirror group, and there was a statistically significant difference (*p* = 0.008) when the measurements made in the follow-up week were compared with the pre-test (Pre: 0.62 ± 0.24; Fu: 1.36 ± 0.63). In the control group, no significant change was observed in all measurements (OSI-API-MLI) (*p* > 0.05) (Table 2).

#### Right athletic single leg static balance test

No time main effect of the right athletic single leg (R-ASL) EC static balance measure on the OSI variable was observed (*F*_3.108_ = 1.196; *p* = 0.315; *η*^2^p = 0.32). However, a time × group interaction was observed (*F*_6.108_ = 19.546; *p* < 0.001; *η*^2^p = 0.521). The source of the difference was evaluated by Bonferroni *Post-Hoc* analysis with a significance level of 0.05: It was determined that the EC static balance OSI value increased significantly (*p* = 0.001) between the pre and post measurements of the mirror group in the group × time interaction, whereas there was no significant difference between the follow-up test and the pre-test (*p* > 0.05). It was found that the EC static balance OSI value decreased between pre and post-measurements (*p* = 0.001) and there was also a statistically significant difference (*p* = 0.002) when the measurements made in the follow-up week were compared with the pre-test (Pre: 3.77 ± 0.99; Fu: 2.50 ± 0.84). There was no time main effect of the R-ASL EC static balance measure on the API variable (*F*_3.108_ = 1.745; *p* = 0.162; *η*^2^p = 0.46). However, a time × group interaction was observed (*F*_6.108_ = 10.408; *p* < 0.001; *η*^2^p = 0.366). The source of the difference was evaluated by Bonferroni *Post-Hoc* analysis with a significance level of 0.05: It was determined that the EC static balance API value increased significantly (*p* = 0.001) between the pre and post-measurements of the mirror group in the group × time interaction, whereas there was no significant difference between the follow-up test and the pre-test (*p* > 0.05). It was determined that the EC Static balance API value decreased between pre- and post-measurements in the group × time interaction of the group without mirror (*p* = 0.001), but there was no significant difference between the follow-up test and the pre-test (*p* > 0.05). No main effect of time for the MLI variable of the R-ASL EC static balance measurement was observed (*F*_3.108_ = 1.137; *p* = 0.338; *η*^2^p = 0.031). However, a time × group interaction was observed (*F*_6.108_ = 7.741; *p* < 0.001; *η*^2^p = 0.301). The source of the difference was evaluated by Bonferroni *Post-Hoc* analysis with a significance level of 0.05: It was determined that the EC static balance MLI value increased significantly (*p* = 0.033) between the pre and post measurements of the mirror group in the group × time interaction, whereas there was no significant difference between the follow-up test and the pre-test (*p* > 0.05). It was found that the EC static balance MLI value decreased between pre and post-measurements (*p* = 0.001) and there was also a statistically significant difference (*p* = 0.002) when the measurements made in the follow-up week were compared with the pre-test (Pre: 2.09 ± 0.64; Fu: 1.35 ± 0.74). In the control group, no significant change was observed in all measurements (OSI-API-MLI) (*p* > 0.05) (Table 3).

**Table 3:** Right Athletic Single Leg Static Balance Test and Left Athletic Single Leg Static Balance Test.

**Eyes closed**		**Mirror zumba group**	**Non-mirror zumba group**	**Control group**	**Right Athletic Single Leg (RASL) Static Balance Test**			
		**Mean ± SD**	**Mean ± SD**	**Mean ± SD**	**Group**	**F**	***p***	*η* ^**2**^ **p**
R-OSI	Pre-test	2.26 ± 1.07	3.76 ± 0.98	3.06 ± 1.41				
	Mid-test	3.07 ± 1.44	2.79 ± 0.83	2.99 ± 1.27	^a^	1.196	0.315	0.32
	Post-test	4.09 ± 1.67^*+^	1.63 ± 0.64	3.03 ± 1.36^*-^	^b^	19.546	**0.001**	0.521
	Follow-up	2.73 ± 1.43	2.5 ± 0.83	3.01 ± 1.25^*+^				
R-API	Pre-test	1.85 ± 1.18	2.74 ± 1.13	2.61 ± 1.39				
	Mid-test	2.4 ± 1.46	1.86 ± 0.56	2.65 ± 1.28	^a^	1.745	0.162	0.46
	Post-test	3.31 ± 1.47^*+^	1.2 ± 0.52	2.63 ± 1.39^*-^	^b^	10.408	**0.001**	0.366
	Follow-up	1.89 ± 0.98	1.89 ± 0.71	2.42 ± 1				
	Pre-test	1.14 ± 0.44	2.09 ± 0.64	1.28 ± 0.54				
	Mid-test	1.58 ± 0.78	1.61 ± 0.72	1.27 ± 0.55	^a^	1.137	0.338	0.031
R-MLI	Post-test	1.68 ± 0.83^*+^	1 ± 0.5^*-^	1.3 ± 0.63	^b^	7.741	**0.001**	0.301
	Follow-up	1.47 ± 0.74	1.34 ± 0.45	1.44 ± 0.64				

					**Left Athletic Single Leg (LASL) Static Balance Test**			
L- OSI	Pre-test	2.5 ± 1.2	3.6 ± 0.98	3.1 ± 1.12				
	Mid-test	3.67 ± 1.67	2.81 ± 0.76	3.13 ± 1.12	^a^	4.469	**0.005**	0.110
	Post-test	3.93 ± 1.48^*+^	1.63 ± 0.83^*-^	3.01 ± 1.04	^b^	14.789	**0.001**	0.451
	Follow-up	2.42 ± 0.87	2.5 ± 0.85^*+^	3.04 ± 1.12				
L- API	Pre-test	1.8 ± 0.85	2.77 ± 0.94	2.51 ± 0.95				
	Mid-test	3.12 ± 1.71	2.09 ± 0.71	2.62 ± 0.86	^a^	3.82	**0.012**	0.96
	Post-test	3.28 ± 1.58^*+^	1.25 ± 0.5^*-^	2.6 ± 0.85	^b^	12.424	**0.001**	0.408
	Follow-up	1.87 ± 0.85	1.8 ± 0.71^*+^	2.56 ± 0.95				
	Pre-test	1.3 ± 0.78	2.02 ± 0.77	1.29 ± 0.75				
	Mid-test	1.5 ± 0.52	1.55 ± 0.47	1.46 ± 0.78	^a^	2.490	**0.064**	0.065
L-MLI	Post-test	1.87 ± 0.58^*+^	0.85 ± 0.38^*-^	1.36 ± 0.76	^b^	9.304	**0.001**	0.341
	Follow-up	1.25 ± 0.36	1.39 ± 0.49^*+^	1.25 ± 0.72				

**Notes.**

a Main effect of time.

b Time × Group interaction.

*+ Statistically significant positive change across pre-test, post-test, and follow-up test.

*- Statistically significant negative change across pre-test, post-test, and follow-up test.

R-OSI Right Overall Stability R-API Right Anterior/Posterior Stability Index R-MLI Right Medial/Lateral Stability Index L-OSI Left Overall Stability L-API Left Anterior/Posterior Stability Index L-MLI Left Medial/Lateral Stability Index

Mean and standard deviation values are presented for each variable. Bold styling indicates statistically significant values.

#### Left athletic single leg static balance test

A time main effect (F3,108=4.469; *p* = 0.005; *η*^2^p = 0.110) and a time ×group interaction (*F*_6.108_ = 14.789; *p* < 0.001; *η*^2^p = 0.451) were observed for the OSI variable of the left athletic single leg (ASL) EC static balance measurement. The source of the difference was evaluated by Bonferroni *Post-Hoc* analysis with a significance level of 0.05: It was determined that the EC static balance OSI value increased significantly (*p* = 0.001) between the pre and post measurements of the mirror group in the group × time interaction, whereas there was no significant difference between the follow-up test and the pre-test (*p* > 0.05). It was found that the EC static balance OSI value decreased between the pre and post-measurements in the group × time interaction of the non-mirror group (*p* = 0.001) and there was a statistically significant difference when the measurements made in the follow-up week were compared with the pre-test (Pre: 3.63 ± 0.98; Fu: 2.51 ± 0.86) (*p* = 0.002). A time main effect (*F*_3.108_ = 3.826; *p* = 0.012; *η*^2^p = 0.96) and a time × group interaction (*F*_6.108_ = 12.424; *p* < 0.001; *η*2p = 0.408) were observed for the API variable of the L-ASL EC static balance measurement. The source of the difference was evaluated by Bonferroni *Post-Hoc* analysis with a significance level of 0.05: It was determined that the EC static balance API value increased significantly (*p* = 0.001) between the pre and post-measurements of the mirror group in the group × time interaction, whereas there was no significant difference between the follow-up test and the pre-test (*p* > 0.05). It was found that the EC static balance API value decreased between the pre and post-measurements in the group × time interaction of the non-mirror group (*p* = 0.001) and there was a statistically significant difference when the measurements made in the follow-up week were compared with the pre-test (Pre: 2.78 ± 0.94; Fu: 1.87 ± 0.73) (*p* = 0.002). No main effect of time for the MLI variable of the L-ASL EC static balance measurement was observed (*F*_3.108_ = 2.490; *p* = 0.064; *η*^2^p = 0.065). However, a time × group interaction was observed (*F*_6.108_ = 9.304; *p* < 0.001; *η*^2^p = 0.341). The source of the difference was evaluated by Bonferroni *Post-Hoc* analysis with a significance level of 0.05: It was determined that the EC static balance MLI value increased significantly (*p* = 0.017) between the pre and post measurements of the mirror group in the group × time interaction, whereas there was no significant difference between the follow-up test and the pre-test (*p* > 0.05). It was found that the EC static balance MLI value decreased between pre and post-measurements (*p* = 0.001) and there was also a statistically significant difference (*p* = 0.002) when the measurements made in the follow-up week were compared with the pre-test (Pre: 2.02 ± 0.77; Fu: 1.39 ± 0.50). In the control group, no significant change was observed in all measurements (OSI-API-MLI) (*p* > 0.05) (Table 3).

#### Right athletic single leg dynamic balance test

In Table 4 there was no time main effect of the R-ASL EC Dynamic balance measure on the OSI variable (*F*_3.108_ = 1.756; *p* = 0.160; *η*^2^p = 0.47). However, a time × group interaction was observed (*F*_6.108_ = 24.020; *p* < 0.001; *η*^2^p = 0.572). The source of the difference was evaluated by Bonferroni *Post-Hoc* analysis with a significance level of 0.05: It was found that the EC Dynamic balance OSI value decreased significantly (*p* = 0.001) between the pre and post measurements of the mirror group in the group × time interaction (*p* = 0.001) and there was a statistically significant difference when the measurements made in the follow-up week were compared with the pre-test (Pre: 3.33 ± 1.02; Fu: 1.96 ± 0.50) (*p* = 0.001). It was found that the EC Dynamic balance OSI value increased significantly (*p* = 0.001) between the pre and post-measurements in the group × time interaction of the non-mirror group, and there was a statistically significant difference (*p* = 0.009) when the measurements made in the follow-up week were compared with the pre-test (Pre: 1.55 ± 0.50; Fu: 2.28 ± 0.91). No time main effect of the R-ASL EC Dynamic balance measure on the API variable was observed (*F*_3.108_ = 0.858; *p* = 0.465; *η*^2^p = 0.023). However, a time × group interaction was observed (*F*_6.108_ = 18.237; *p* < 0.001; *η*^2^p = 0.503). The source of the difference was evaluated by Bonferroni *Post-Hoc* analysis with a significance level of 0.05: It was found that the EC Dynamic balance API value decreased significantly (*p* = 0.001) between the pre and post-measurements of the mirror group in the group × time interaction (*p* = 0.001) and there was a statistically significant difference when the measurements made in the follow-up week were compared with the pre-test (Pre: 2.71 ± 0.78; Fu: 1.54 ± 0.66) (*p* = 0.001). It was found that the EC Dynamic balance API value increased significantly (*p* = 0.001) between the pre and post-measurements in the group × time interaction of the non-mirror group and there was a statistically significant difference (*p* = 0.009) when the measurements made in the follow-up week were compared with the pre-test (Pre: 1.25 ± 0.34; Fu: 1.95 ± 0.91). No time main effect of the R-ASL EC Dynamic balance measure on the MLI variable was observed (*F*_3.108_ = 1.120; *p* = 0.344; *η*^2^p = 0.030). However, a time ×group interaction was observed (*F*_6.108_ = 5.248; *p* < 0.001; *η*^2^p = 0.226). The source of the difference was evaluated by Bonferroni *Post-Hoc* analysis with a significance level of 0.05: It was found that the EC Dynamic balance MLI value decreased significantly (*p* = 0.013) between the pre and post measurements of the mirror group in the group × time interaction, and a statistically significant difference (*p* = 0.003) when the measurements made in the follow-up week were compared with the pre-test (Pre: 1.56 ± 0.79; Fu: 0.95 ± 0.47). No significant change was observed in the EC Dynamic balance MLI value between the pre and post-measurements in the non-mirror group in the group × time interaction when the measurements made in the follow-up week were compared with the pretest (*p* > 0.05). In the control group, no significant change (OSI-API-MLI) was observed in all measurements (*p* > 0.05) (Table 4).

**Table 4:** Right Athletic Single Leg Dynamic Balance Test and Left Athletic Single Leg Dynamic Balance Test.

**Eyes closed**		**Mirror zumba group**		**Non-mirror zumba group**	**Control group**	**Right Athletic Single Leg (RASL) Dynamic Balance Test**			
		**Mean ± SD**	**Mean ± SD**	**Mean ± SD**	**Group**	**F**	***p***	*η* ^**2**^ **p**
	Pre-test		3.32 ± 1.02	1.54 ± 0.5	3.2 ± 2.3				
	Mid-test		2.35 ± 0.67	2.17 ± 0.69	3.15 ± 2.39	^a^	1.756	0.160	0.47
R-OSI	Post-test		1.31 ± 0.33^*-^	2.91 ± 1.33^*+^	3.1 ± 2.34	^b^	24.020	**0.001**	0.572
	Follow-up		1.96 ± 0.49^*+^	2.28 ± 0.91^*-^	3.15 ± 2.3				
R-API	Pre-test		2.71 ± 0.77	1.25 ± 0.34	2.64 ± 1.93				
	Mid-test	1.95 ± 0.75	1.69 ± 0.64	2.7 ± 2.04	^a^	0.858	0.465	0.023
	Post-test	1.07 ± 0.36^*-^	2.3 ± 1.07^*+^	2.78 ± 2.27	^b^	18.237	**0.001**	0.503
	Follow-up	1.53 ± 0.65^*+^	1.95 ± 0.9^*-^	2.63 ± 2.22				
R-MLI	Pre-test		1.56 ± 0.79	0.85 ± 0.3	1.47 ± 1.54				
	Mid-test	1.07 ± 0.41	1.19 ± 0.51	1.51 ± 1.36	^a^	1.120	0.344	0.030
	Post-test	0.66 ± 0.31^*-^	1.45 ± 0.64	1.24 ± 0.78^*+^	^b^	5.248	**0.001**	0.226
	Follow-up	0.95 ± 0.46^*+^	1 ± 0.31	1.48 ± 1.18				

						**Left Athletic Single Leg (LASL) Dynamic Balance Test**			
	Pre-test	4.34 ± 3	1.78 ± 0.76	3.7 ± 1.98				
L-OSI	Mid-test	2.57 ± 0.81	2.19 ± 0.7	3.69 ± 2.06	^a^	1.913	0.132	0.50
	Post-test	1.41 ± 0.57^*-^	3.13 ± 1.32	3.65 ± 1.99	^b^	9.258	**0.001**	0.340
	Follow-up	2.62 ± 2.13^*^	2.4 ± 1.05	3.61 ± 1.82				
L-API	Pre-test	2.72 ± 1.42	1.27 ± 0.44	2.83 ± 1.23				
	Mid-test	2.36 ± 1.73	1.66 ± 0.49	3.18 ± 1.63	^a^	0.453	0.715	0.012
	Post-test	1.02 ± 0.46^*-^	2.43 ± 1.1^*+^	3.06 ± 1.72	^b^	6.700	**0.001**	0.271
	Follow-up	2.12 ± 2.27	1.94 ± 0.9	2.89 ± 1.5				
L-MLI	Pre-test	2.5 ± 2.73	1 ± 0.76	1.9 ± 1.7				
	Mid-test	1.42 ± 0.56	1.26 ± 0.5	1.83 ± 1.23	^a^	1.060	0.369	0.032
	Post-test	1.02 ± 0.61^*-^	1.55 ± 0.57	1.83 ± 1.19	^b^	3.358	**0.005**	0.157
	Follow-up	1.11 ± 0.45*	1.33 ± 0.53	2.01 ± 1.53				

**Notes.**

a Main effect of time.

b Time × Group interaction.

*+ Statistically significant positive change across pre-test, post-test, and follow-up test.

*- Statistically significant negative change across pre-test, post-test, and follow-up test.

R-OSI Right Overall Stability R-API Right Anterior/Posterior Stability Index R-MLI Right Medial/Lateral Stability Index L-OSI Left Overall Stability L-API Left Anterior/Posterior Stability Index L-MLI Left Medial/Lateral Stability Index

Mean and standard deviation values are presented for each variable. Bold styling indicates statistically significant values.

#### Left athletic single leg (asl) dynamic balance test

No main effect of time for the OSI variable of the L-ASL EC dynamic balance measurement was observed (*F*_3.108_ = 1.913; *p* = 0.132; *η*^2^p = 0.50). However, a time × group interaction was observed (*F*_6.108_ = 9.258; *p* < 0.001; *η*^2^p = 0.340). The source of the difference was evaluated by Bonferroni *Post-Hoc* analysis with a significance level of 0.05:In the group × time interaction of the mirror group, it was found that the EC Dynamic balance OSI value decreased between pre and post-measurements (*p* = 0.001) and there was a statistically significant difference when the measurements made in the follow-up week were compared with the pre-test (Pre: 4.34 ± 3.01; Fu: 2.63 ± 2.14) (*p* = 0.009). No significant change was observed in the EC Dynamic balance OSI value between the pre and post-measurements in the non-mirror group in the group × time interaction when the measurements made in the follow-up week were compared with the pretest (*p* > 0.05). In the control group, no significant change was observed in all measurements (OSI-API-MLI) (*p* > 0.05). No time main effect of the L-ASL EC Dynamic balance measure on the API variable was observed (*F*_3.108_ = 0.453; *p* = 0.715; *η*^2^p = 0.012). However, a time × group interaction was observed (*F*_6.108_ = 6.700; *p* < 0.001; *η*^2^p = 0.271). The source of the difference was evaluated by Bonferroni *Post-Hoc* analysis with a significance level of 0.05: It was determined that the EC Dynamic balance API value decreased significantly (*p* = 0.001) between the pre and post-measurements of the mirror group in the group × time interaction, whereas there was no significant difference between the follow-up test and the pre-test (*p* > 0.05). It was determined that the EC Dynamic balance API value increased significantly (*p* = 0.008) between the pre and post-measurements in the group × time interaction of the non-mirror group, whereas there was no significant difference between the follow-up test and the pre-test (*p* > 0.05). No time main effect of the L-ASL EC Dynamic balance measure on the MLI variable was observed (*F*_3.108_ = 1.060; *p* = 0.369; *η*^2^p = 0.0329). However, a time × group interaction was observed (*F*_6.108_ = 3.358; *p* < 0.001; *η*^2^p = 0.157). The source of the difference was evaluated by Bonferroni *Post-Hoc* analysis with a significance level of 0.05: It was found that the EC Dynamic balance MLI value decreased significantly (*p* = 0.034) between the pre and post measurements of the mirror group in the group × time interaction, and a statistically significant difference (*p* = 0.034) when the measurements made in the follow-up week were compared with the pre-test (Pre: 2.51 ± 2.73; Fu: 1.11 ± 0.45). No significant change was observed in the EC Dynamic balance MLI value between the pre and post-measurements in the non-mirror group in the group × time interaction when the measurements made in the follow-up week were compared with the pretest (*p* > 0.05). In the control group, no significant change was observed in all measurements (OSI-API-MLI) (*p* > 0.05) (Table 4).

**Figure 4:**
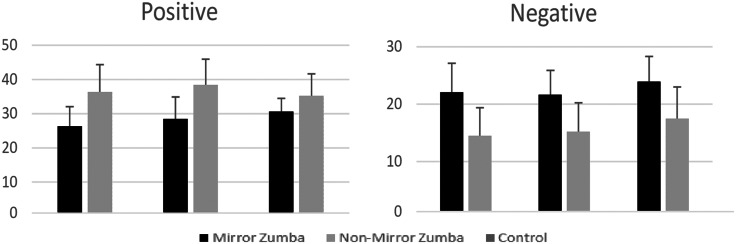
Positive–Negative Mood State (PANAS) scale pre-test-post-test results.

Although no statistically significant differences were found between the groups, similar trends were observed across all three. In the mirror group, mean positive affect scores increased (Pre: 26.21 ± 5.83; Post-test (St): 36.21 ± 8.15), while negative affect scores decreased (Pre: 22 ± 5.13; St: 14.43 ± 4.94). A similar pattern emerged in the non-mirror group, with an increase in positive affect (Pre: 28.38 ± 6.49; St: 38.31  ± 7.6) and a decrease in negative affect (Pre: 21.62 ± 4.25; St: 15.15 ± 5.1). Interestingly, the control group also exhibited a comparable trend, with an increase in positive affect (Pre: 30.5 ± 3.94; St: 35.17 ± 6.48) and a decrease in negative affect (Pre: 23.92 ± 4.38; St: 17.42 ± 5.6). These findings suggest a general tendency toward improved mood states across all participants, regardless of the experimental intervention. This overall trend may not be solely attributable to the Zumba sessions or mirror use, but could also reflect the influence of non-experimental factors such as the social environment, group dynamics, or broader contextual elements—such as seasonal mood variation or fluctuations in emotional well-being throughout the academic calendar (Fig. 4).

## Discussion

In this study, it was hypothesized that mirror use would negatively affect joint position sense and dynamic balance, while having a positive effect on static balance. Furthermore, mirror use was expected to have no significant effect on mood state. However, the findings did not fully support this assumption. While the mirror group showed a deterioration in proprioceptive (joint position sense (JPS)) accuracy and an improvement in static balance, the expected negative effect on dynamic balance was not evident and consistent across all measures (see Tables 2 and 4). However, some conditions, especially in follow-up tests, were associated with worsening or maintenance of dynamic balance measures in participants trained in the mirror condition. This contradiction suggests that mirror feedback can have both positive and negative consequences depending on the type of motor tasks performed: in static positions, it can help the individual to realize and maintain the correct position and balance (visuospatial alignment), while in proprioceptive-based dynamic tasks, it can become an element that can weaken the interaction with the individual’s internal sensory signals (attention, muscle tension, joint position, *etc.*). This result may cause temporary decreases in balance control and movement accuracy. Therefore, these findings are in line with previous studies that show that the effects of mirror use on body awareness and motor control may vary according to task type (Radell et al., 2014; Diehl, 2016). Accordingly, the partial discrepancy between the hypothesis and the results reveals the complex and context-specific nature of visual feedback in action-based learning.

### Inclinometer (Joint Position Sense)

In this study, significant improvements were observed in the right and left knee flexion parameters at 15°, 30°, 60°, and 90° in both the mirror and non-mirror training groups. A significant effect of time was identified in the measurement of knee joint flexion in the mirror group. Additionally, the time × group interaction revealed that joint position sense decreased significantly in the mirror group between pre-and post-tests, whereas the non-mirror group showed significant improvement. These findings suggest that receiving Zumba dance training in a mirrored environment negatively affects knee joint position sense (JPS). The follow-up test results conducted five weeks after the completion of the Zumba training showed that the non-mirror group-maintained improvements in right 15°–60° and left 15° JPS, while the mirror group continued to exhibit deterioration in right and left JPS at 15°, 30°, 60°, and 90°. These results are consistent with the literature. In their study comparing mirror and non-mirror training, Dearborn & Ross (2006), found that mirror-based training supported the acquisition and retention of difficult movements, whereas individuals who learned without mirrors, though they learned more easily, tended to forget what they had learned more quickly. Thus, this suggests that even if the learning outcome results in a disadvantage, it may still be maintained over time. The follow-up results showed that despite initial improvements, the non-mirror group began to regress, whereas the mirror group retained its statistically significant difference.

There is limited research on the effects of exercise, training, or physical activity performed in mirror environments on proprioception. However, this study’s findings align with those reported in the existing literature. Diehl (2016), a dancer and scholar, questioned the presence of mirrors in dance studios in her review article. She emphasized that mirrors can be beneficial in certain learning contexts but may negatively affect movement sensation and joint position sense. According to Diehl (2016), reliance on external cues (*e.g.*, teacher, audience, mirrors) can lead individuals to distrust their internal sensory experience, significantly reducing their ability to feel and experience movement. Similarly, Brodie & Lobel (2008), argued that excessive reliance on visual cues may interfere with the ability to attend to kinesthetic information and hinder reliance on proprioceptive feedback. Proprioception, defined as the ability to perceive the body’s position and movement, plays a critical role in athletic performance and physical well-being (Yılmaz et al., 2024). Mirrors undoubtedly contribute to self-monitoring by providing feedback on posture, alignment, and movement. For instance, mirrors help determine whether a leg is straight or if the back is upright or rounded. However, in visually dominant learning environments, such feedback may overshadow proprioceptive processing (Oliver, 2008).

Mirrors should be viewed as educational instructional tools, and their use should be carefully considered (Radell et al., 2014). For example, when focusing on proprioceptive awareness, mirrors should be used selectively, and learners should eventually be encouraged to train without mirrors once initial learning is achieved. Professor Sally Radell has led research in this field at Emory University for over a decade. In her studies, which examine mirrors as a visual tool in teaching and learning, she emphasized that while mirrors may facilitate technical skill development, they may simultaneously hinder body image, performance, and proprioception due to the interrelated nature of these components. Students who viewed mirrors as helpful for skill acquisition also reported experiences of body objectification, as they constantly compared their physical selves with the mirrored image (Radell et al., 2021), although mirrors helped promote technical development, they often disrupted students’ concentration and prevented them from fully engaging their kinesthetic and proprioceptive senses (Radell et al., 2014). For example, when students see their chin too close to their chest in the mirror, they shift their kinesthetic awareness to the chin, adjust the position, and recheck the mirror. This initiates a repeated cycle of “look, feel, look again”, resulting in a fragmented process of visual correction and proprioceptive adjustment. As Ehrenberg (2010), noted, this cyclical disruption of kinesthetic awareness may contribute to a decline in JPS.

### Biodex (Static-Dynamic Balance)

Disruptions in mechanoreceptor function are believed to impair balance by contributing to proprioceptive deficits (Radell, Adame & Cole, 2002). As balance depends on the integrated function of visual, vestibular, and proprioceptive systems (Daneshjoo et al., 2012), proprioceptive development is often supported through balance training (Hanney, 2000; Malliou et al., 2004; Hutt & Redding, 2014). In this study, participants in the mirror group showed significant improvements in static balance (measured *via* the eyes-closed Postural Stability Test (PST) and Athletic Single Leg Test (ASL)), while dynamic balance significantly declined. Conversely, the non-mirror group exhibited reduced static balance but significant improvement in dynamic balance. In the study conducted by Waer et al. (2024a), Waer et al. (2024b) and Waer et al. (2024c), it was reported that a 12-week Zumba exercise program implemented with female participants significantly improved balance performance. These findings align with literature suggesting that mirrors support static balance Notarnicola et al. (2014), and that structured Zumba training contributes to dynamic balance development (Donath et al., 2014).

Previous studies indicate that mirrors may facilitate technical skill acquisition in dance but may also reduce proprioceptive awareness (Brodie & Lobel, 2008; Radell et al., 2014). Excessive reliance on visual feedback can disrupt kinesthetic focus and diminish bodily self-awareness (Brodie & Lobel, 2008; Oliver, 2008). In our study, mirror-group participants focused more on their external appearance than the technical execution of movements, which may have negatively affected their dynamic balance. In contrast, non-mirror participants, who concentrated more on correct movement execution, showed better proprioceptive outcomes. Similar observations were reported by Coker (2016) and Anderson (2011), who emphasized that although mirrors can facilitate learning, they may simultaneously distance learners from their internal bodily perception. In conclusion, while mirror-based training may contribute to improvements in static balance, it may adversely affect dynamic balance and proprioceptive sensitivity.

### PANAS (Mood Scale)

Contrary to these findings, several studies in the literature have reported negative psychological effects associated with mirror use. Radell, Adame & Cole (2002), found that participants trained with mirrors exhibited lower body image scores. Similarly, Ehrenberg (2010) and Diehl (2016), suggested that mirrors could contribute to emotional distress and distortions in body perception. Martin Ginis, Jung & Gauvin (2003), reported that sedentary women exercising in mirrored environments experienced declines in mood and self-efficacy, attributing this to increased self-awareness and body image concerns.

On the other hand, some studies found no adverse emotional effects related to mirror use. Chmelo et al. (2009), observed no significant differences in emotional responses during resistance training with or without mirrors, while Cameron (2015), reported no notable changes in self-presentation or affect among novice exercisers. Lamarche, Gammage & Strong (2009) and Frayeh & Lewis (2018), noted that although social physique anxiety increased in mirrored environments, this could be more closely related to the nature of the group exercise rather than mirror exposure. Despite conflicting evidence, the present study suggests that mirror use during Zumba training does not produce significant negative effects on participants’ moods.

According to the findings of this study, there was no statistically significant change in positive or negative affect among participants who received Zumba training in front of a mirror. Similarly, no significant affective change was observed in the non-mirror group. However, mean scores indicate an increase in positive mood and a decrease in negative mood in both experimental groups. Interestingly, a similar trend was also observed in the control group, with an increase in positive affect and a decrease in negative affect, despite the absence of Zumba training. This suggests that the observed affective improvements may not be solely attributable to Zumba participation or the use of mirrors, but could also be influenced by general physical activity engagement, group dynamics, or other contextual factors affecting emotional well-being. Therefore, while the findings confirm that mirror use during Zumba does not negatively impact mood, they also highlight the potential contribution of non-experimental variables to affective outcomes

## Limitations

Although our research provides important findings, it also has some limitations that should be taken into consideration in future studies. First of all, the sample consisted only of physically inactive female university students aged 18-25 years, and it may be of interest to apply it to populations of different age groups or with varying levels of physical activity. Second, although all tests were performed in both eyes-open and eyes-closed conditions, only data from the eyes-closed condition were analyzed, limiting the holistic assessment of the effect of visual feedback on proprioceptive performance. Also, the environmental factors of the hall used in the mirrored environment (lighting, mirror placement, individual perception differences, etc.) were not fully standardized, which may have had an impact on the results. On the other hand, in future research, it would be interesting to measure other functional performance parameters such as upper extremity strength, flexibility, and muscular endurance to evaluate our results in more detail. The fact that only the PANAS scale was used in the evaluation of mood change limits the interpretation of psychological effects; the fact that other psychological variables such as body perception, self-efficacy, or motivation towards exercise were not taken into account narrows the scope of the study. Also, one of the prominent limitations of this study is that the effect (as measured by the PANAS) was only assessed at two time points (pre-test and post-test). However, other variables were evaluated at four different times. This limited time resolution limits our ability to make a comprehensive assessment of how sensory responses change, particularly with follow-up period or interim test performance. Finally, although the follow-up test administered at week 15 provides information in terms of short-term persistence, the lack of longer-term follow-up limits inferences about the sustainability of training effects. Moreover, the observation that the control group exhibited similar changes in mood—namely, increased positive affect and decreased negative affect—compared to the experimental groups complicates the interpretation of the specific emotional effects of the intervention (*i.e.,* Zumba training and mirror use). This raises the possibility that mood changes may not be solely attributable to the experimental conditions but may also be influenced by external factors such as seasonal psychological variation, general social interaction within the university setting, or academic calendar-related stress fluctuations. Future studies would benefit from a more controlled design that accounts for these potential confounding variables.

## Conclusion

This study found that mirror use during Zumba training negatively affected proprioception and dynamic balance but improved static balance. In contrast, non-mirror training enhanced proprioception and dynamic balance but reduced static balance. Mirror use may hinder kinesthetic learning despite aiding visual feedback. Mood changes were observed in both groups but were not statistically significant, possibly due to participants’ sports backgrounds. In conclusion, mirror use in Zumba dance training appears to negatively affect proprioception and dynamic balance, while positively influencing static balance and showing no significant effect on mood state. Although participants were classified as physically inactive based on the absence of regular, structured exercise in the past six months, it is important to note that they were all students of the Faculty of Sport Sciences. This academic background likely contributed to a basic familiarity with physical activity, sports-related discourse, and body awareness. Therefore, despite their recent physical inactivity, their prior exposure to sport may have played a protective role—particularly in buffering against potential negative body image responses in a mirrored exercise environment. It is recommended that mirror use be applied with caution, particularly in programs aiming to enhance proprioceptive awareness and internal bodily perception. Future studies should include more diverse participant groups across various age and experience levels, employ longitudinal designs, and explore psychological factors such as self-efficacy, body image, and social anxiety in greater depth.

## Practical applications

The findings of this study provide important insights for instructors, trainers, and movement educators who utilize mirror-based environments in group exercise settings such as Zumba. While mirrors may help facilitate visual feedback and enhance technical precision in the early stages of motor learning, prolonged reliance on them may hinder proprioceptive development and dynamic balance. For programs that aim to improve bodily awareness, joint position sense, and kinesthetic control, instructors are advised to reduce mirror dependency over time and encourage participants to focus on internal cues. In this context, gradually transitioning from mirrored to mirror-free practice may support deeper engagement with movement sensations and foster kinesthetic learning. Alternating between mirrored and non-mirrored sessions could offer a balanced approach, supporting skill acquisition and sensory-motor integration. Furthermore, practitioners should be mindful of individual differences, particularly among beginners, and adjust visual feedback strategies accordingly. These results may also inform curriculum planning in physical education, dance instruction, and therapeutic exercise contexts where bodily self-awareness and movement control are key learning outcomes.

##  Supplemental Information

10.7717/peerj.20453/supp-1 Supplemental Information 1 Data

## Abbreviations

API: Anterior–Posterior Stability Index
BMI: Body Mass Index
D-API: Dynamic Anterior-Posterior Stability Index
D-MLI: Dynamic Medial-Lateral Stability Index
D-OSI: Dynamic Overall Stability Index
EC: Eyes Closed
L-API: Left Anterior/Posterior Stability Index
L-MLI: Left Medial/Lateral Stability Index
L-OSI: Left Overall Stability
MLI: Medial–Lateral Stability Index
OSI: Overall Stability Index
PANAS: The Positive and Negative Affect Schedule
PST: Postural Stability
R-API: Right Anterior/Posterior Stability Index
R-MLI: Right Medial/Lateral Stability Index
R-OSI: Right Overall Stability
S-API: Static Anterior-Posterior Stability Index
SD: Standard deviation
S-MLI: Static Medial-Lateral Stability Index
S-OSI: Static Overall Stability Index

## Additional Information and Declarations

### Data Availability

The following information was supplied regarding data availability: The data is in the Supplemental File.
